# Synergistic Assembly of 1DZnO and Anti-CYFRA 21-1: A Physicochemical Approach to Optical Biosensing

**DOI:** 10.34133/bmef.0064

**Published:** 2024-09-18

**Authors:** Rafael A. Salinas, Shirlley E. Martínez Tolibia, Patricia G. Zayas-Bazán, Sandra E. Rodil, Mathew T. Mathew, Andrés Navarrete, Guillermo Santana, Ateet Dutt

**Affiliations:** ^1^ Departamento de Materiales de Baja Dimensionalidad, Instituto de Investigaciones en Materiales Universidad Nacional Autónoma de México, C.P. 04510, Mexico City, México.; ^2^ Department of Biomedical Science, UIC College of Medicine, Rockford, IL 61107, USA.; ^3^ Facultad de Química, Departamento de Farmacia, Universidad Nacional Autónoma de México, Ciudad Universitaria C.P. 04510, Coyoacán, Mexico City, México.

## Abstract

**Objective:** We conducted a comprehensive physicochemical analysis of one-dimensional ZnO nanowires (1DZnO), incorporating anti-CYFRA 21-1 immobilization to promote fast optical biomarker detection up to 10 ng ml^−1^. **Impact Statement:** This study highlights the effectiveness of proof-of-concept 1DZnO nanoplatforms for rapid cancer biomarker detection by examining the nanoscale integration of 1DZnO with these bioreceptors to deliver reliable photoluminescent output signals. **Introduction:** The urgent need for swift and accurate prognoses in healthcare settings drives the rise of sensitive biosensing nanoplatforms for cancer detection, which has benefited from biomarker identification. CYFRA 21-1 is a reliable target for the early prediction of cancer formation that can be perceptible in blood, saliva, and serum. However, 1DZnO nanostructures have been barely applied for CYFRA 21-1 detection. **Methods:** We assessed the nanoscale interaction between 1DZnO and anti-CYFRA 21-1 antibodies to develop rapid CYFRA 21-1 detection in two distinct matrices: PhosphateBuffered Saline (PBS) buffer and artificial saliva. The chemical modifications were tracked utilizing Fourier transform infrared spectroscopy, while transmission electron microscopy and energy dispersive spectroscopy confirmed antigen–antibody interplay over nanostructures. **Results:** Our results show high antibody immobilization efficiencies, affirming the effectiveness of 1DZnO nanoplatforms for rapid CYFRA 21-1 testing within a 5-min detection window in both PBS and artificial saliva. Photoluminescence measurements also revealed distinct optical responses across biomarker concentrations ranging from 10 to 1,000 ng ml^−1^. **Conclusion:** Discernible PL signal responses obtained after 5 min affirm the potential of 1DZnO nanoplatforms for further advancement in optical biomarker detection for application in early cancer prognosis.

## Introduction

**Fig. 1.**
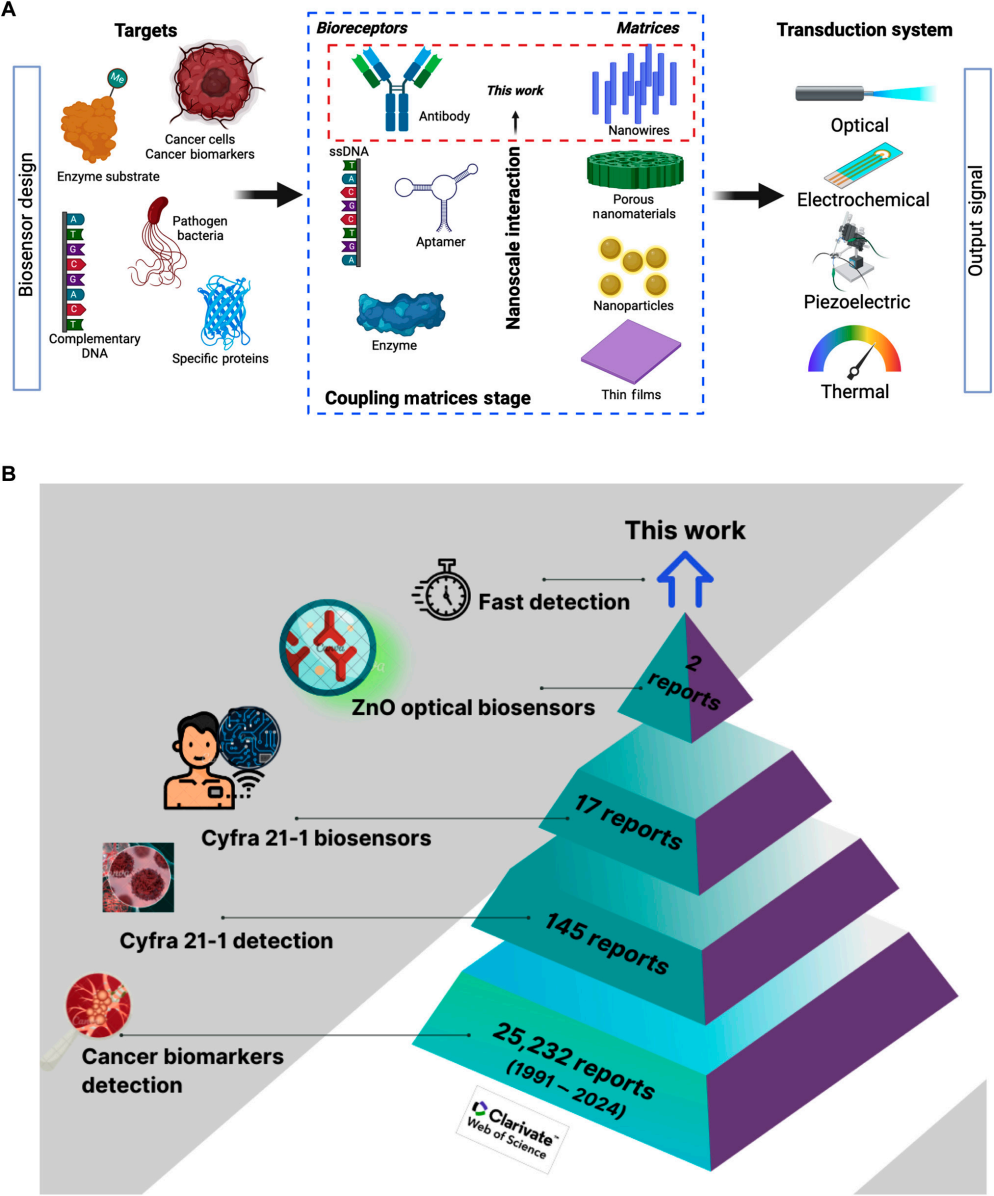
(A) Schematic overview of the principal elements involved in biosensors design and development (created with biorender.com). (B) Current research trends on CYFRA 21-1 biodetection (Web of Science Database, 2024).

Cancer is a persistent worldwide disease that has triggered a substantial economic burden on healthcare systems, with nearly 10 million deaths all over the world (World Health Organization, 2020) [1]. A current description of the macroeconomic costs of cancer treatments from 144 countries has estimated a projection cost of 25.2 trillion dollars from 2020 to 2050, which denotes the need for investment in early diagnostic technologies [2]. Disease progression is monitored by sequential stages of tumor differentiation, classified into 4 grades, and generally detected by microscopic visualization at advanced stages when symptoms have appeared [3]. Cancer diagnostics is primarily based on lab testing such as cytogenic analysis, complete blood count, blood chemistry, tumor marker tests [4], urine cytology [5], and radiology [6], and depending on the cancer type, imaging and biopsies can provide more details about tumor size, location, and severity in real time [7,8]. Nevertheless, there is a limitation in their extended use because of the expensive infrastructure and specialized equipment required for frequent testing, with a more complicated implementation in low- and middle-income countries (LMICs) [9] due to the lack of suitable screening programs in public health services, poor organization, affordability of screening tests, adequate infrastructure, appropriate cost-effectiveness, and cost–benefit analyses to be carried out, among others [10]. Thus, the importance of prognosis, diagnosis, and constant monitoring of cancer biomarkers can determine successful detection and treatment [11] and represent a step ahead for developing early detection technologies such as biosensors. In this regard, these devices can evolve into efficient diagnostic methods that could contribute to the early detection of cancer in healthcare facilities, reducing the economic burden caused by expensive oncological treatments. This suggestion is supported by an evaluation of priorities in cancer research directed to solve problems in LMICs, where primary prevention strategies, early and constant screening for cancer control, and adoption of technologies such as point-of-care diagnostics (lab-on-a-chip), telemedicine, image analysis, and pattern recognition for pathologies must be implemented in public health services [12,13]. With respect to cancer biomarkers research, it has been found that lung cancer, biliary tract cancer, breast and bladder cancers, oral squamous cell carcinoma (OSCC), and other epithelial tumors can express a common biomarker known as CYFRA 21-1, related to the cytokeratin 19 fragment expressed in cytoskeleton filaments from epithelial cells [14–17]. CYFRA 21-1 can serve as a biomarker for cancer prediction perceptible in blood samples [17], saliva, and serum in patients with OSCC at minimum concentrations [18,19], and it is usually detected by biochemical quantification in plasma samples, immunoassays, and other hybridization methods [20]. However, emerging alternatives consider the design of electrochemical, photoelectrochemical, or optical biosensors that can contribute to cancer diagnosis, improving their sensitivity using nanomaterials [21]. Biosensing platforms for cancer biomarker detection are mainly fabricated of gold, graphene, metal-oxide semiconductors, carbon nanotubes, indium tin oxide, glass–carbon and other composites, which offer improved sensitivity within the order of μg ml^−1^ to pg ml^−1^. In particular, electrochemical biosensors generally use a combination of materials such as AuNPs, Si_3_N_4_/MoS_2_, Au electrode, SnS_2_/SnS/Bi_2_S_3_, and ZnCdS@ NPC-ZnO for the fabrication of electrodes, coatings, and coupling materials for enhancing output signal responses [22,23]; however, this could unbalance the final cost of biosensor devices. Therefore, it is desired to use versatile nanomaterials of high precision that offer advantages of low cost, easy handling, and suitable biosensing performance in complex samples at low concentrations [24]. One feasible option is ZnO-based nanomaterials that offer outstanding semiconductive and optical properties such as controllable photoluminescence (PL) emission, high surface-to-volume ratio, high isoelectric point [25–27], and even antimicrobial activity against pathogenic bacteria [28,29]. Yet, the design and conceptualization of biosensors involve a multidisciplinary approach to achieve a complete biosensor device (Fig. 1A), where the first stage comprises the bioreceptors’ design based on target biomolecules. This work involves precisely this stage for coupling bioreceptors that identify oral cancer biomarker CYFRA 21-1, establishing a basis for further developing precise and sensitive biosensors.

From a current search in the Web of Science database, the optical properties of ZnO nanostructures have been barely applied for cancer biomarker detection. The research tendency on CYFRA 21-1 biomarker detection (Fig. 1B) demonstrates the interest in the development of biosensors (17 articles), but only a few reports have developed ZnO-based biosensors targeting CYFRA 21-1 [30,31]. These works have used ZnO nanomaterials in different combined forms, as in the case of ZnCdS@NPC-ZnO used for photoelectrochemical immunosensors with a limit of detection (LOD) of a few pg/ml [30], or combined with carbon quantum dots (CQDs/ZnO nanocomposite) to form a sensitive immunofluorescent system that reaches an LOD of less than a few ng/ml [31].

**Fig. 2.**
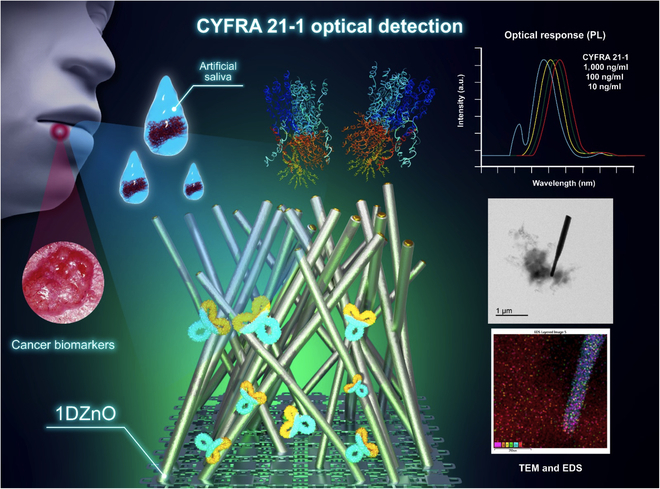
Schematic view of the experimental techniques followed to study the nanoscale interaction between anti-CYFRA 21-1 bioreceptors over 1DZnO nanoplatforms.

Hence, a distinct opportunity exists to advance the development of prognostic devices capable of continuously monitoring suspected cancer cases. Additionally, saliva-based biosensors are emerging as feasible alternatives for diagnosing systemic diseases and biomarkers associated with cancer and other metabolites. Their appeal lies in their noninvasive approach to sample collection and processing [32]. Thus, in this work, we propose the use of 1DZnO nanoplatforms to determine the physical and chemical basis of the nanoscale interaction between 1DZnO and anti-CYFRA 21-1 to assess the rapid detection of the CYFRA 21-1 biomarker in 2 distinct matrices: phosphate-buffered saline (PBS) buffer and artificial saliva (Fig. 2). The advantages offered by 1DZnO nanoplatforms are reduced contact times (5 min), low-cost material (single ZnO noncomposite), improved optical response by intrinsic fluorescence, low fabrication costs (without composite material or electrode), and stability.

## Results

**Fig. 3.**
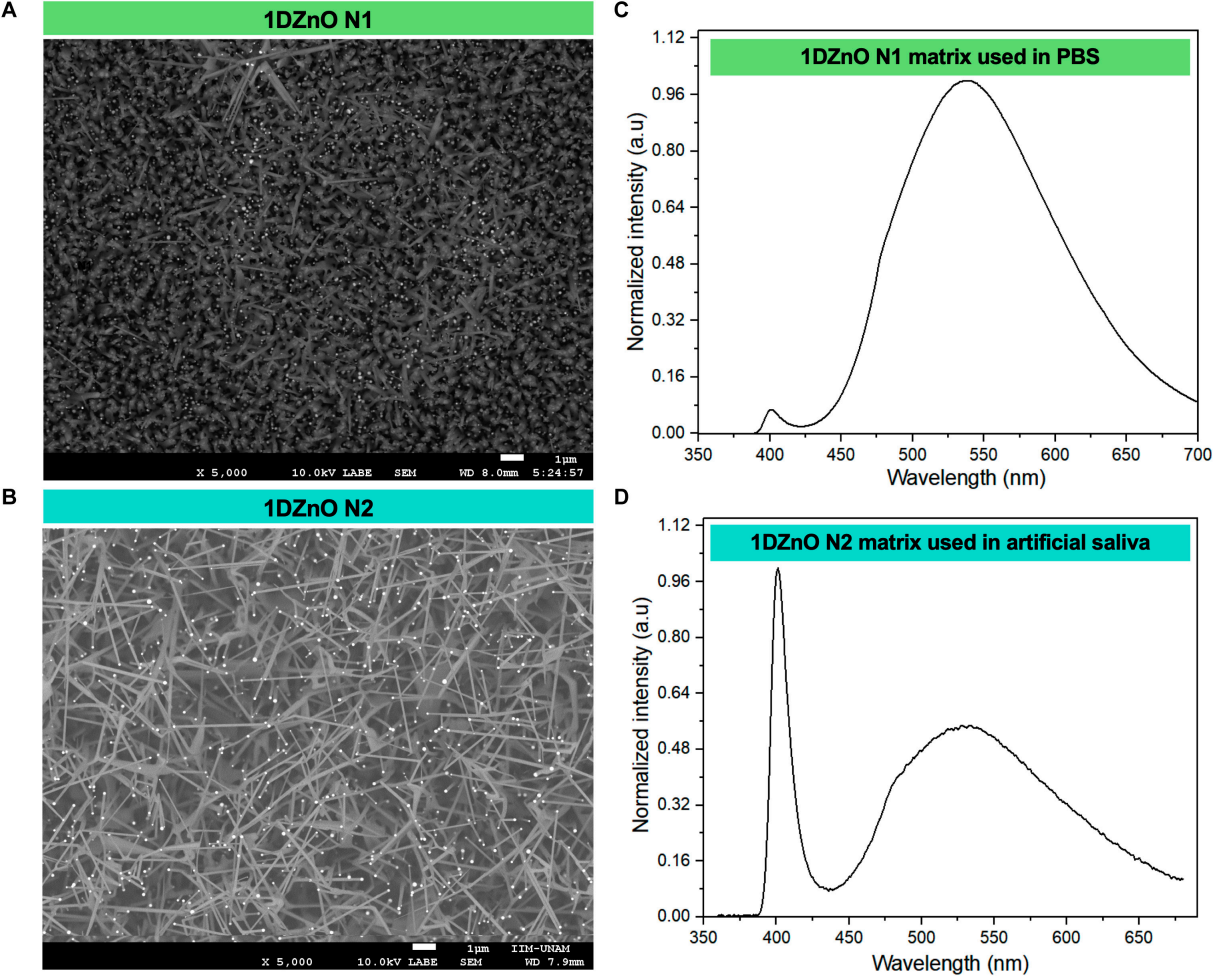
Characterization of 1DZnO samples (N1 and N2). SEM micrographs of synthesized nanostructures (A and B) and their corresponding PL spectra (C and D).

Currently, there are different strategies for nanomaterials’ growth compatible with diverse biological applications, such as hydrothermal methods [33,34] (or vapor–liquid–solid [VLS] techniques). In this work, the proposed nanoplatforms involve the metal catalyst growth of 1DZnO nanostructures over Si substrates, as studied in previous works [35,36], since this system allows for better homogeneity control of nanostructures. After synthesizing the samples using VLS, we chose 2 different characteristic morphologies of ZnO nanowires as observed by scanning electron microscopy (SEM). The sample set N1 showed combined morphologies of short flakes and nanowires of approximated dimensions 1.53 ± 0.447 μm length and 105 ± 26.4 nm diameter (Fig. 3A). In contrast, the sample set N2 presented the most homogeneous morphologies of thin and large nanowires of dimensions 2.74 ± 0.824 μm length and 124 ± 21.5 nm diameter and a higher density on Si substrates (Fig. 3B).

The alterations in nanostructured morphology observed here correspond to fluctuations in the optical properties and emission mechanisms of 1DZnO. Our prior research indicates that the PL response of 1DZnO can be tailored by adjusting the observed emission bands [37]. Furthermore, we have demonstrated that leveraging various 1DZnO morphologies and regulating their emission offers benefits in designing and manufacturing nanoplatforms for optical transduction-based nanobiosensors [38].

This feature was observed in the PL spectra (Fig. 3C and D), which correspond to the morphologies N1 and N2, respectively. The first PL band refers to the excitonic recombination near-band emission (NBE) at 400 nm, whereas the second is related to the permitted states within the forbidden gap generated by intrinsic defects in the ZnO structure known as deep-level emission (DLE) band approximately at 540 nm. Notable distinctions were observed between NBE and DLE, which offered intriguing insights into the effects of chemical surface modification during biofunctionalization. Specifically, 1DZnO N1 exhibits a high DLE and a low NBE band, indicating elevated oxygen defects and surface vacancies [27]. Conversely, 1DZnO N2 exhibits a high NBE band, attributed to a robust excitonic peak at 400 nm, indicating fewer oxygen defects and, consequently, a diminished DLE band. Thus, we aimed to correlate alterations in the optical output signal under controlled conditions in 1 mM PBS buffer with those observed in a complex matrix (artificial saliva) for biomarker detection, as detailed in the subsequent sections.

### Monitoring of chemical surface modifications by FTIR spectroscopy

**Fig. 4.**
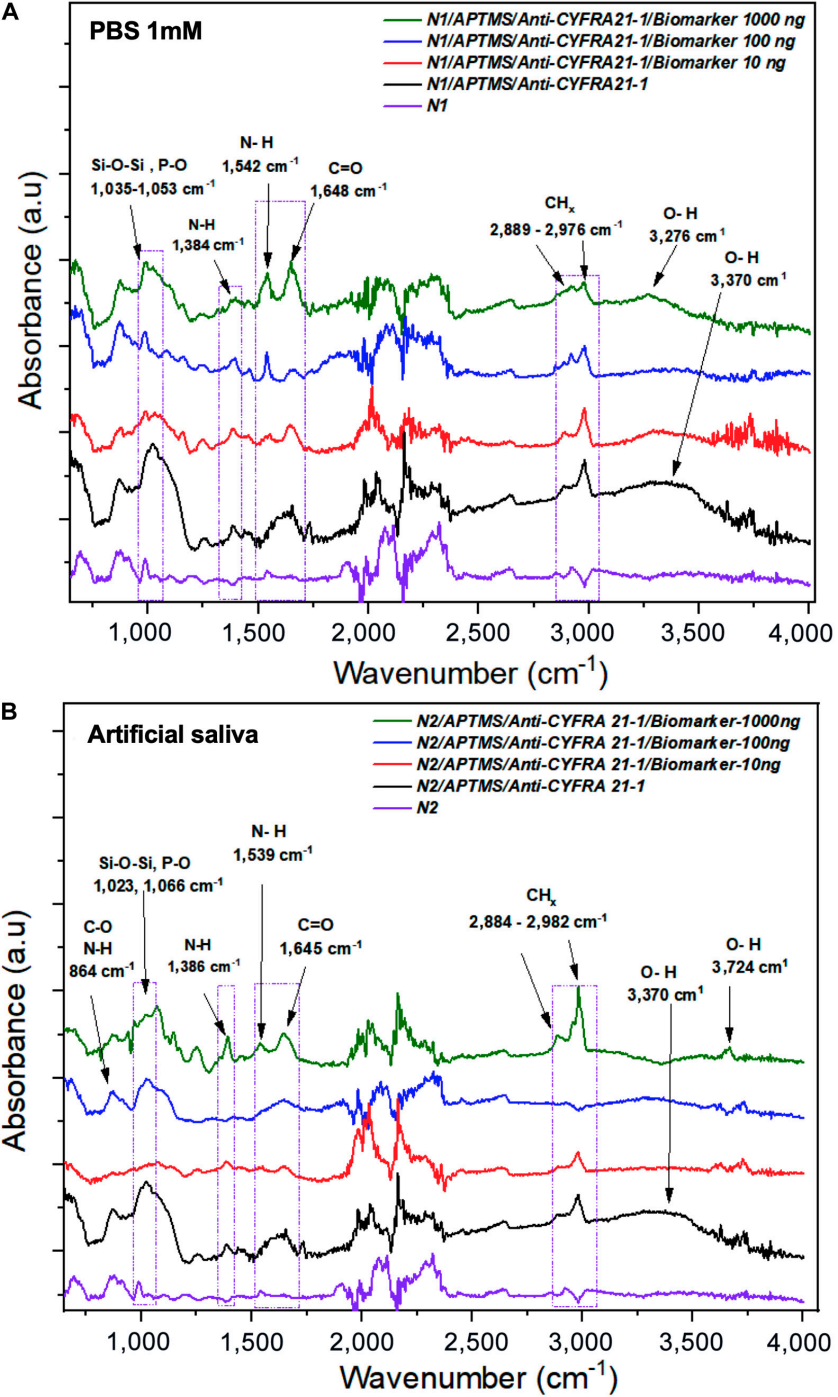
FTIR spectroscopy of surface chemical modification, antibody immobilization, and CYFRA21-1 detection. (A) FTIR spectra of N1 nanoplatforms and biomarker detection in PBS, and (B) FTIR spectra of N2 nanoplatforms and biomarker detection in artificial saliva.

Fourier transform infrared (FTIR) spectroscopy is a versatile and nondestructive technique employed to follow up chemical modification during 1DZnO nanoplatform biofunctionalization and biomarker detection. The spectroscopic analysis for detection at different concentrations (10, 100, and 1,000 ng ml^−1^) is shown in this section, including controls as a reference. CYFRA 21-1 detection assays were performed in 1 mM PBS using N1 nanoplatforms (Fig. 4A). Nanostructures without chemical modification allowed us to contrast functional groups appearing after biodetection stages. The spectrum after biofunctionalization with 3-Aminopropyltrimethoxysilane (APTMS) is N1/APTMS/anti-CYFRA21-1 that corresponds to antibody immobilization, and shows signal peaks related to amide I at 1,648 cm^−1^ and amide II at 1,542 cm^−1^ corresponding to C=O and N-H, respectively. In addition, the signals associated with the functionalization, such as Si-O-Si (1,035 cm^−1^) and C-H, O-H from 2,889 to 3,276 cm^−1^, show differences after contact with antibodies and detection of CYFRA 21-1 (as a function of concentration). Since the biomarker is also a protein, the observed changes in the spectra are expected to occur in the same regions as in bioreceptors. For instance, amide I and amide II regions have a better definition with biomarker increasing concentration, allowing us to distinguish that the protein is recognized over the nanostructure surface. In addition, we observed the increase of overtones from C-H bonds related to the amino acids at the region 2,850 to 2,976 cm^−1^. The 1,000 ng ml^−1^ CYFRA 21-1 spectra showed higher intensity and constructive interference signals.

On the other hand, detection in complex matrices is a primary objective in developing targeted application devices. Therefore, we proposed using artificial saliva to perform CYFRA 21-1 detection assays using N2 nanoplatforms, from which the corresponding FTIR analysis was performed at the same concentrations (10, 100, and 1,000 ng ml^−1^), including controls before and after the biofunctionalization process (Fig. 4B). Analogous to the previous spectra, signals related to the CYFRA 21-1 biomarker were observed, which have slight shifts compared to the peaks previously described in PBS buffer (1DZnO N1) but are associated with the vibrational modes of the same functional groups. Notably, the rapid detection process (5 min) enabled precise observation of amide I and II regions without interference from salts in the artificial saliva.

However, pinpointing new signals via FTIR remains challenging due to overlapping characteristic peaks between the detected biomarker and bioreceptor, both proteins. Therefore, the observed signals are a constructive interference to the protein region at C=O (1,645 cm^−1^) and C-H (2,889 to 2,976 cm^−1^), most perceptible at 1,000 ng ml^−1^ with higher intensity. As an additional comparison to assess the stability of immobilization and biomarker detection, we obtained the comparative FTIR spectra of 1DZnO nanostructures with anti-CYFRA 21-1 immobilization 6 months later (red) and after recent antibody immobilization (black) (Fig. S1A). Both spectra allowed us to observe characteristic signals of amide 1 (C = O), amide 2 (N-H), and disulfide bridges from antibodies (C-S and S-S), confirming the main signals of proteins. The storage time slightly modifies the O-H and P-O group ratio due to the preservation in the buffer; however, the stability of 1DZnO nanoplatforms after several months is remarkable and outstanding. These results can support the immobilization strategy’s stability and the fact that the devices could be stored during this time, with the antibodies available for biomarker recognition. Furthermore, the devices that were kept in contact with CYFRA 21-1 at 10 and 1,000 ng/ml were also characterized, showing the characteristic protein signals from amides 1 and 2, as well as C-S contributions from methionine amino acids (Fig. S1B and C). The presence of the same FTIR signals after 6 months strongly supports the preservation of 1DZnO biofunctionalized nanoplatforms before and after contact with the target biomarker.

This extends the shelf life of functional 1DZnO nanoplatforms and provides an advantage for confirming stability after and before detection, even if 1DZnO is stored for prolonged periods. However, to identify lower concentrations accurately, the FTIR spectroscopy technique requires more complex and systematic analyses, and signals have proved challenging to discern during this preliminary characterization. From these results, we aimed to carry out a microscopic examination by robust and accurate techniques that could elucidate the physical and chemical interaction between nanostructures and anti-CYFRA 21-1 antibodies and provide details about how it is accomplished during biomarker detection in PBS buffer and artificial saliva, as shown in the following section.

### Analysis of CYFRA21-1 detection by TEM in PBS and artificial saliva

**Fig. 5.**
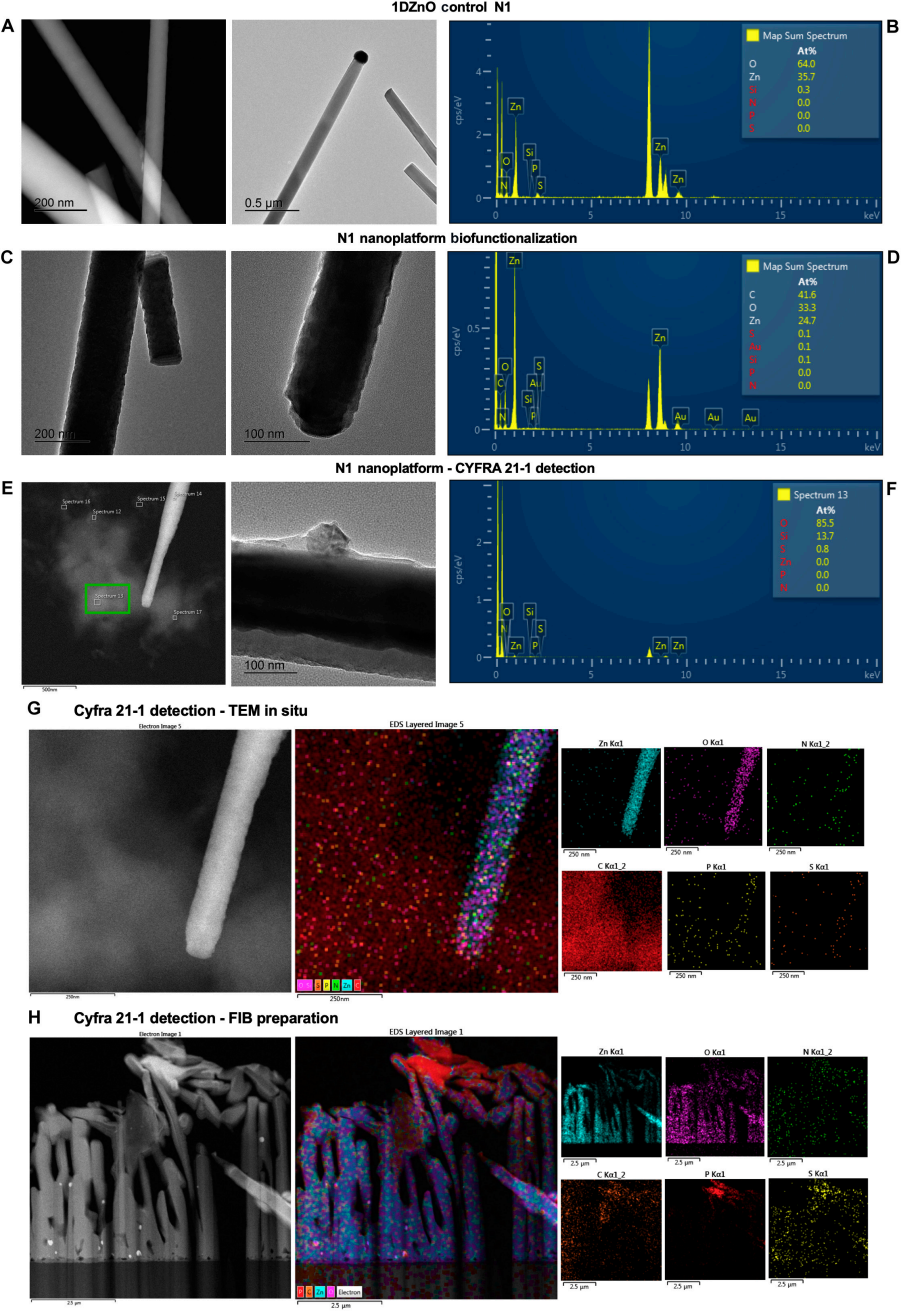
Microscopic visualization of 1DZnO N1 nanoplatforms’ nanostructural conformation after biofunctionalization and CYFRA 21-1 detection in PBS buffer. (A) TEM images of bare N1, (B) EDS analysis of bare N1, (C) TEM images of biofunctionalization, (D) EDS analysis of biofunctionalization, (E) TEM images of biomarker detection, (F) EDS analysis of detection, (G) compositional mapping by EDS in STEM mode, and (H) FIB micromachining and compositional mapping by EDS.

We conducted analyses using transmission electron microscopy (TEM) and prepared samples using focused ion beam (FIB) to investigate nanoscale interactions at the interface between nanomaterials and attached biomolecules (such as antibody–protein complexes). First, the control N1 nanostructures are illustrated, showcasing their characteristic morphology alongside the Au tip from the VLS synthesis method (Fig. 5A). Notably, the nanowires exhibit polished surfaces without noticeable deformations resulting from chemical modifications. This was corroborated by energy dispersive spectroscopy (EDS) analyses, which revealed a distinct signal solely from precursor elements Zn and O originating from the nanomaterial synthesis (Fig. 5B and Fig. S2). On the other hand, the biofunctionalized N1 with antibodies showed a general modification of morphology on the nanowires, observed by the visible roughness on its surface because of the generated self-assembly monolayers (SAMs) from the APTMS reaction (Fig. 5C), as previously described by the authors [38]. A monolayer forms as a thin envelope on the surface. Detection by EDS (Fig. 5D) shows the presence of C, O, Zn, and Si in the higher proportion corresponding to biofunctionalization and antibody attachment, demonstrating the morphological changes observed before (Fig. 5C and Fig. S3). Following CYFRA 21-1 detection, the presence of protein aggregates on the surface of 1DZnO nanowires was observed, along with a cloud-like structure attached to the nanowire (Fig. 5E and Fig. S4).

Since cytokeratins’ molecular weight falls within the range of 40 to 68 kDa, the formation of large protein complexes of the CYFRA 21-1 biomarker, visible via TEM, is suggested. Additional micrographs offer perspectives where these protein aggregates are distinctly visible (Fig. S4). Moreover, the corresponding EDS analysis reveals the presence of sulfur (S) originating from disulfide bridges within the CYFRA 21-1, alongside a higher prevalence of oxygen (O) from its amino acids and silicon (Si) from APTMS (Fig. 5F).

**Fig. 6.**
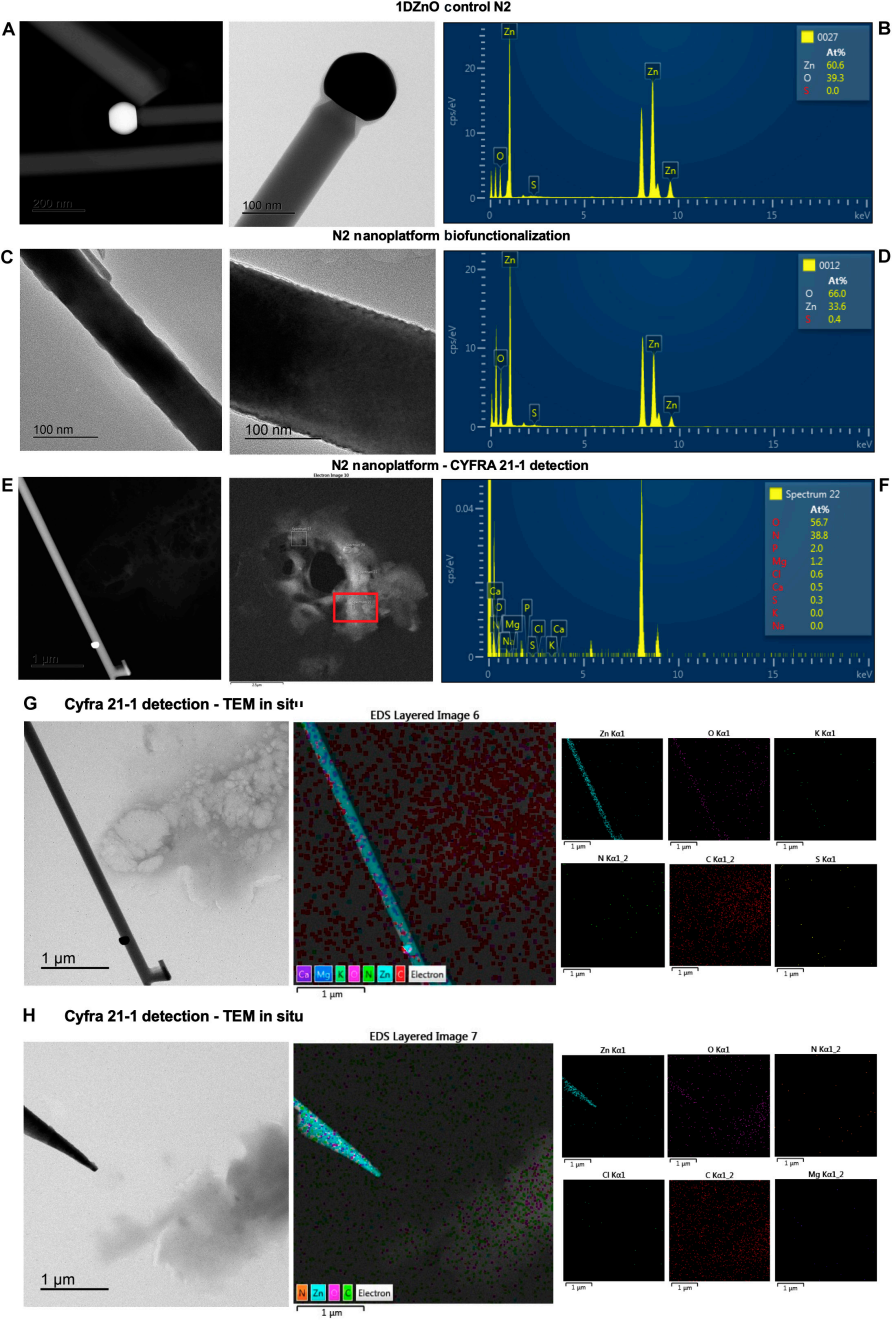
Microscopic visualization of 1DZnO N2 nanoplatforms’ nanostructural conformation after biofunctionalization and CYFRA 21-1 detection in artificial saliva. (A) TEM images of bare N2, (B) EDS analysis of bare N2, (C) TEM images of biofunctionalization, (D) EDS analysis of biofunctionalization, (E) TEM images of CYFRA 21-1 detection, (F) EDS analysis of CYFRA 21-1 detection, and (G and H) compositional mapping by EDS in STEM mode.

These observations further support the compositional analysis conducted via EDS (Fig. 5G). Here, Zn and O elements are identified as constituting the nanowire, while carbon (C), oxygen (O), nitrogen (N), phosphorus (P), and sulfur (S) are detected on both the surface of 1DZnO and within the cloud-like complex. The heightened presence of carbon is particularly notable, indicating the abundance of organic biomolecules. Furthermore, an FIB preparation from a micromachine cross-section is presented to analyze a different region of N1 nanoplatform, in which the elemental scanning determined that Zn and O are part of the nanostructures. In contrast, C, N, S, and P are located on the surface as part of the attached biomolecules (Fig. 5H). Nanoscale interactions were also evaluated after biofunctionalization and CYFRA 21-1 detection in artificial saliva samples. It is shown in the nanostructural analysis of control N2 nanoplatforms, in which the polished morphology of ZnO nanowires and the only presence of Zn and O are observed (Fig. 6A and B). After biofunctionalization, an increased roughness on the nanowire surface was visually detected, as described before for N1 nanoplatforms (Fig. 6C and D). Subsequently, upon detection of the CYFRA 21-1 biomarker, we observed the formation of large protein complexes immobilized on the surfaces of 1DZnO N2, persisting as cohesive aggregates following exposure to artificial saliva (Fig. 6E). The corresponding EDS analysis (Fig. 6F) proved that protein complexes show the presence of O, N, P, and S in higher amounts. In contrast, Mg, Cl, and Ca were also detected, corresponding to the mineral salt remains from artificial saliva composition. Details on the elemental mapping of N2 nanoplatforms after biomarker detection allowed us to observe 2 regions where nanowires can recognize protein complexes formed between antibodies and CYFRA 21-1 (Fig. 6G and H). The compositional mapping validates these observations, revealing a notable abundance of elements such as C, O, N, and S, indicative of proteinaceous biomolecules, alongside Mg, Cl, and K associated with artificial saliva. Based on these findings, further experimental work is warranted to determine sensitivity, selectivity, and detection limits, which are crucial for advancing the development of ZnO-based nanobiosensor devices for detecting cancer biomarkers.

After the obtained results about chemical modifications and the formation of protein complexes were determined by FTIR and TEM, we proposed PL spectroscopy as the optical transduction system, which could allow the complete evaluation of the detection performance of 1DZnO nanoplatforms to determine the possible influence of nanostructure interaction, interferences from matrices (PBS and artificial saliva), and antibody immobilization efficiencies. The output signal responses obtained by PL measurements are presented in the next section.

### Overall performance of N1/N2 nanoplatforms for optical biomarker detection

An essential consideration for optical devices is the ability to distinguish transduction signals from blank controls. In this regard, PL offers the advantage of rapid response and high sensitivity, minimizing interference from other components in the sample that can be specific and compatible with implementation on large-scale fabrication devices [39,40].

**Fig. 7.**
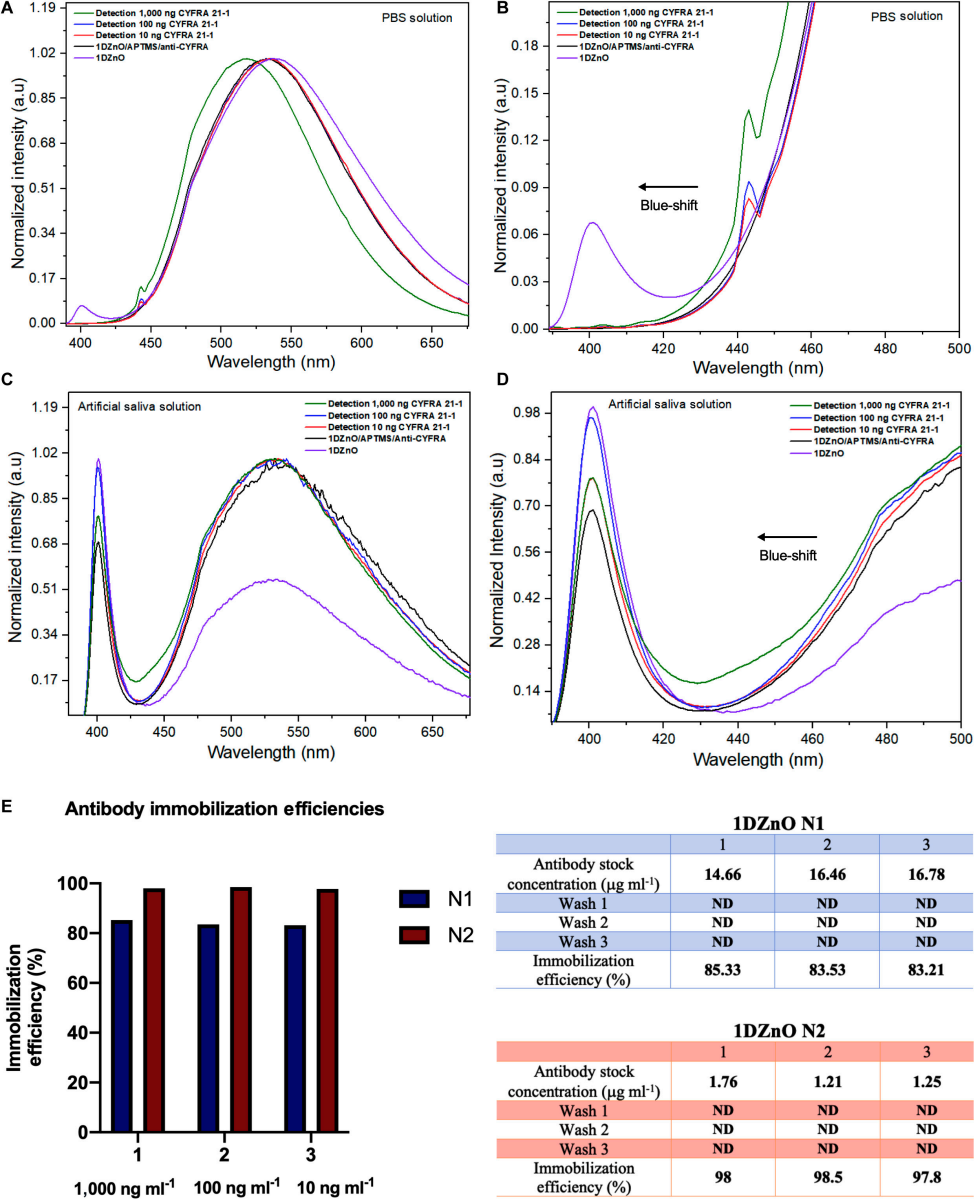
PL spectra of CYFRA 21-1 biomarker detection at different concentrations (A) in 1 μM PBS buffer (1DZnO N1), (B) zoomed-in view from 1DZnO N1, and (C) in artificial saliva (1DZnO N2), (D) zoomed-in view from 1DZnO N2. (E) Antibody immobilization efficiencies obtained after biofunctionalization of N1 and N2 nanoplatforms. ND means nondetectable.

In the case of 1DZnO, PL response is a property applicable to biosensing, albeit currently constrained to cancer biomarker detection. Hence, the analysis of PL response demonstrated discernible changes related to rapid CYFRA 21-1 detection (5 min) using 1DZnO nanoplatforms across concentrations of 10, 100, and 1,000 ng ml^−1^ under controlled conditions in both PBS buffer and artificial saliva (Fig. 7). The control emission bands of N1 (purple line) were obtained from the initial PL characterization (Fig. 7A), where low NBE and high DLE are present, as described previously (Fig. 3C). When antibody immobilization occurs (black), we can detect a shift in DLE and a quenching effect at the NBE band. Since a SAM with APTMS was formed during biofunctionalization, as confirmed by FTIR analysis (Fig. 4A) and TEM micrographs (Fig. 5C), the chemical modifications could contribute to generating higher interstitial zinc and oxygen vacancies (V_o_Zn_i_), which can impact the intensity of the NBE band (quenching) [38,41]. In addition to the chemical process, most of the CYFRA 21-1 antibodies can cover the surface, as observed microscopically by FIB preparation and compositional mapping (Fig. 5H and Fig. S5), contributing to the shift view in DLE [42]. Subsequently, upon analyzing N1 nanoplatforms post-detection, varying concentrations of the CYFRA 21-1 biomarker induce a shift in DLE, possibly linked to the distribution of protein aggregates on the surface (formed between antibodies and the CYFRA 21-1 biomarker), as could be notably appreciated by TEM (Fig. 5E), resulting in a blue-shift emission. This suggests that higher biomarker concentrations lead to more remarkable emission changes from the principal radiative center, accompanied by a minor signal emerging at 443 nm, not evident in the controls. This phenomenon is highlighted by zooming into the 400- to 500-nm region associated with the target presence (Fig. 7B), accentuated as biomarker concentrations rise at 1,000 ng ml^−1^. It is hypothesized that protein complexes may introduce interference in the PL spectra wavelength. Then, we performed a PL evaluation after CYFRA 21-1 detection in artificial saliva using N2 nanoplatforms (Fig. 7C). The control PL spectra showed a higher NBE band contribution and a lower DLE band (purple), as observed by the single nanomaterial (Fig. 3D). After biofunctionalization, a substantial increase in the signal intensity in DLE above the control emission is noticed, which suggests contributions from protein biomolecules (antibodies) derived from charge interactions between nanostructures and the protein complexes, as reported before by Liu et al. [43] and also observed by TEM (Fig. 6E), with a consequent reduction of NBE (black). Following CYFRA 21-1 detection in saliva, noticeable blue shifts and overall signal modifications were observed relative to biomarker concentration, with a sustained elevation in the DLE band profile. A close-up view between the 400- and 500-nm region revealed discernible differences in the NBE band (Fig. 7D). These results are supported by the obtained scanning tunneling electron microscopy (STEM) images (Fig. 6G and H), where the formation of big protein complexes is highly associated with the interaction between antibodies and CYFRA 21-1. The reduced intensity of NBE in N2 is attributed to the biofunctionalization process. However, the subsequent intensity recovery is linked to residual mineral salts from artificial saliva adhered to the nanostructure surface, potentially influencing signal modification through adhesion or aggregate formation (Fig. S6). Previous studies have reported a fluorescence emission peak at 405 nm in human saliva, supporting our observations [44]. The capability of the proposed 1DZnO nanoplatforms to distinguish low concentrations (ng) has its explanation in the bioselective layer generated from antibodies. The high immobilization efficiency of bioreceptors is always desired and strongly influences biosensing devices’ sensitivity. Accordingly, we determined antibody immobilization efficiencies by Bradford assays post-biofunctionalization, underscoring the importance of an optimized biofunctionalization approach to achieve consistently high immobilization efficiencies (80% to 97%) across N1 and N2 nanoplatforms (Fig. 7E).

From these results, it is established that the proposed 1DZnO nanobiosensors offer an LOD of 10 ng ml^−1^, which is in accordance with the cancer risk levels of CYFRA 21-1 reported by diverse works, where concentrations above 3.5 ng/ml and increased values near 12 ng/ml indicate cancer disease condition [45]. In addition, clinical tests established that in patients with OSCC, the values in saliva vary between 17.46 ±1.46 ng/ml [46] and 14.03 to 35.04 ng/ml [19].

As observed, N2 nanoplatforms showed the highest immobilization efficiencies (97% to 98%), which could contribute to the differentiated response obtained in a complex matrix such as artificial saliva, surpassing the effects of interferences. The high efficiencies are probably strongly related to the formation of protein complexes observed by STEM and EDS and could also play an essential role in the sensitivity of optical output signals measured by PL.

## Discussion

The advancements in cancer identification and treatment technologies have been remarkable in recent years. However, substantial efforts are required to invest in novel technologies that facilitate rapid prognostics, early detection, and continuous monitoring of cancer threats. One promising avenue is the development of nanobiosensors. Our focus lies in creating biosensing devices tailored for timely cancer detection in complex matrices, thus setting a precedent for future commercial applications. To accomplish this goal, a detailed analysis must account for nanoscale interactions between nanomaterials and biomolecules. Considering this, we advocate for utilizing microscopic techniques in our study to gain insights into these nanoscale interactions, establishing a solid groundwork for future characterizations. As observed from the results, the functional groups observed by FTIR suggested that surface chemical modification was carried out, triggering a high presence of proteins (antibody-target). On the other hand, TEM confirmed the immobilization, the presence of the biomarker, and the possible interferences from the experimental matrices (PBS salts and artificial saliva). Accordingly, the efficacy of TEM/FIB/EDS techniques was demonstrated for the characterization of protein complexes of CYFRA 21-1 firmly attached even over single nanowires, as determined in other works [47]. PL discernible changes finally confirmed observations after biomarker detection at different concentrations. However, it must be mentioned that complex matrices such as artificial saliva can preserve noticeable disparities in PL spectra, where achieving a discernible response between concentrations without interference effects is paramount. Therefore, a focused examination of physical and chemical interactions at the nanoscale promises to yield fresh insights and invaluable knowledge for enhancing the optical response of ZnO nanobiosensors. While our proof-of-concept design featuring 1DZnO-based nanoplatforms showcases promise, further experiments are warranted to determine reproducible LODs and selectivity, thus presenting a comprehensive proposal for optical nanobiosensors in cancer biomarker detection. Leveraging labeled biomarkers could greatly enhance our understanding through extensive real-time interaction studies and protein complex formation analysis, aiding in determining nanobiosensor sensitivity.

 In conclusion, this study underscores the pivotal role of nanostructure morphologies from 1DZnO in PL responses. Nanostructure density, length, and oxygen defects highly influence optical responses during wet chemistry for biofunctionalization. In addition, PL spectroscopy emerged as a sensitive technique capable of identifying low concentrations of the CYFRA 21-1 biomarker within short contact times (5 min), even in the presence of complex matrices like artificial saliva. It is noteworthy that our implemented biofunctionalization strategy showed high efficiencies of antibody immobilization (83% to 98%) in all nanoplatforms, which explains that optical detection revealed a discernible PL signal response after just 5 min of contact time in both matrices, affirming the potential of 1DZnO nanoplatforms for further advancement in optical biomarker detection. Nevertheless, assessing the optical detection performance of 1DZnO nanoplatforms in saliva obtained from clinical samples will be imperative in future experiments to evaluate potential interferences from other proteins or enzymes that might impede the optical PL signal response.

As shown in this work, the development of efficient biodetection strategies faces different challenges, starting from the efficient immobilization of bioreceptors to the nanomaterial platforms, which must ensure their stability before contact with the target, under different storage and testing conditions (pH variations, temperature, and long-term storage). In addition, efficient quantifiable response signal (transduction system), precision, and reproducibility along variable target concentrations are some of the most considerable points to be solved [24] before proving testing in complex matrices or clinical samples. For the specific detection of CYFRA 21-1, the future perspectives must consider the biosensing performance of testing devices in samples of different natures, such as saliva, human serum, and liquid biopsy samples, which must provide a robust response despite the interferences. Precisely, robust output signal and sensitive response are the gold standard that must be reached for reproducible detection of cancer biomarkers in both configurations (optical and electrochemical) without vastly overrating the final cost of biosensor devices. As these issues can be solved, the feasibility of biosensors will become a reality for medical interventions, not only for detection in sick patients but also for early cancer screening in healthy populations.

## Materials and Methods

### Experimental and technical design

This study elucidates the impact of the fabrication process on biofunctionalized 1DZnO nanoplatforms and their optical efficacy in detecting the CYFRA 21-1 biomarker under controlled conditions in both PBS and artificial saliva. To accomplish this, 1DZnO nanostructures were synthesized, biofunctionalized, and characterized by microscopic and spectroscopic techniques for further investigating the physicochemical interactions at the nanoscale using a suite of techniques further described in the following subsections, as depicted in Fig. 2.

### Nanomaterial synthesis and characterization

As described in previous reports, 1DZnO nanostructures were synthesized by a metal catalyst-assisted vapor phase growth method over Si substrates [38]. Nanostructure morphologies were characterized by SEM images obtained with JEOL JSM-7600F equipment (using backscattered electrons at a magnification of 5,000×).

### Biofunctionalization of 1DZnO nanoplatforms and characterization

The biofunctionalization process was performed as described in previous works [38]. Briefly, surface activation of 1DZnO was made by an initial hydroxylation with 0.1M KOH diluted in methanol for 10 min, followed by a washing step with methanol (5 min). Then, the silanization process was performed by using a 1% APTMS solution (Sigma-Aldrich) dissolved in acetone, submerging the hydroxylated samples in this solution for 5 min, rinsing with acetone, and placing them under thermal treatment at 115 °C ± 1 °C for 90 min. Subsequently, antibody immobilization was conducted with 100 μg ml^−1^ polyclonal anti-CYFRA 21-1 antibody solution, in which 1DZnO biofunctionalized samples were placed at 4 °C for 60 min. Then, samples were rinsed with water thrice (5 min each) to avoid unspecific adhesions.

Monitoring of nanoplatforms’ chemical modifications was performed by FTIR spectroscopy in the mode of attenuated total reflectance by using a JASCO 4X equipment in the middle region (400 to 4,000 cm^−1^).

### CYFRA 21-1 detection assays

The CYFRA 21-1 biomarker (76 μg ml^−1^) was used as the protein component for detection experiments. A stock concentration of 1,000 ng ml^−1^ was prepared from the primary solution in 1 mM PBS buffer and artificial saliva, and then serial dilutions were made to obtain 100 and 10 ng ml^−1^ for nanoplatform detection assays. 1DZnO nanoplatforms were placed at each dilution (300 μl) during 5 min of contact time, and subsequently, they were rinsed 3 times and preserved in sterile water (500 μl) for further validations. The detailed composition of the artificial saliva used was methyl 4-hydroxybenzoate (2 g l^−1^), carboxymethylcellulose (2 g l^−1^), CaCl_2_ 2H_2_O (0.625 g l^−1^), MgCl_2_ 6H_2_O (0.059 g l^−1^), CaCl_2_ (0.166 g l^−1^), K_2_HPO_4_ (0.804 g l^−1^), and KH_2_PO_4_ (0.326 g l^−1^).

### FIB and compositional mapping by EDS

FIB micromachining and STEM were used as advanced structural techniques for nanoscale screening. FIB JEOLl JEM-9320FIB equipment (Ga: source, 30 kV) was used to prepare the samples; additionally, Pt deposition was performed to enhance TEM resolution. Moreover, a lamella of micrometric dimensions was obtained to perform an advanced TEM study to obtain compositional screening to confirm the presence of organic molecules. Compositional mapping by EDS in STEM mode measurements was carried out using JEOL ARM-200F microscope equipment to evaluate protein complexes attached after detection.

### PL measurements

The optical response signal is essential to determine the relationship between the surface modification of 1DZnO nanoplatforms and their interaction with targets to establish the optical changes and how the emission spectrum of the material has been modified before and after detection. A Spectra Pro 2500i spectrometer and a Kimmon Koha He-Cd laser (325 nm/6 mW) were used to measure the emission spectra of the samples.

As described, we used 1 mM PBS buffer and artificial saliva as solvent matrices for biomarker dilutions to analyze the biosensing response and performance under controlled conditions.

### Antibody immobilization efficiency

To confirm the number of antibodies attached to the surface of 1DZnO nanoplatforms, the antibody concentrations were quantified by the Bradford protein assay (Bio-Rad) in a microplate reader (Bio-Rad Model 680). A calibration curve was plotted from different concentrations of BSA protein standard (Bio-Rad) at an interval between 0 and 25 μg ml^−1^ (Fig. S7). Data were fitted by linear regression to obtain the equation correlating the UV absorbance measurement (OD 595 nm) with the protein concentration. Protein quantification from antibody stocks before and after immobilization (washes) was performed to obtain the antibody immobilization efficiencies.

## Data Availability

Additional data related to this paper may be requested from the corresponding author.
