# Supplementary material for: Synergistic Assembly of 1DZnO and Anti-CYFRA 21-1: A Physicochemical Approach to Optical Biosensing
Source: BME Front. 2024 Sep 18;5:0064. doi: 10.34133/bmef.0064 (PMC11408934; doi:10.34133/bmef.0064)
Supplement: Supplementary 1 — Figs. S1 to S7 [file bmef.0064.f1.zip › Supplementary information.docx]

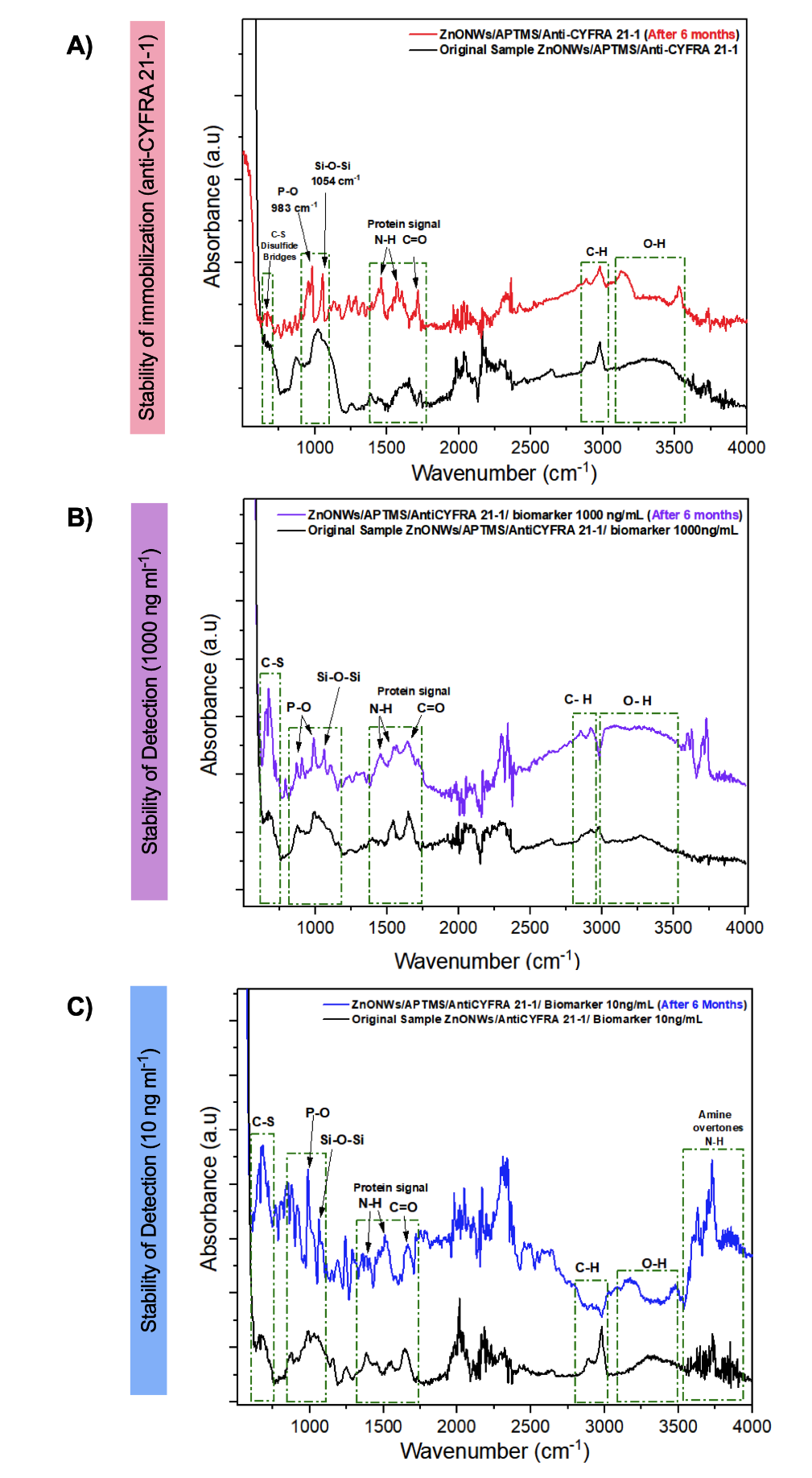
**SUPPLEMENTARY MATERIAL**

**Fig. S1.** FTIR spectra showing the stability of biofunctionalized 1DZnO nanoplatforms before (A) and after biomarker detection of 1000 ng ml^-1^ B) and 10 ng ml^-1^ C). A comparison between recent and preserved samples after 6 months at 4˚C is provided.

**1DZnO control nanostructures**


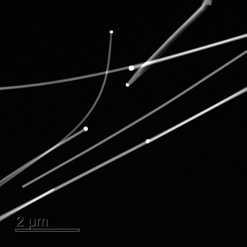

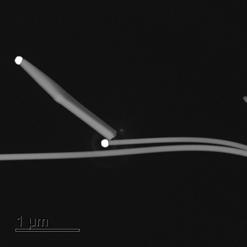


**Fig. S2.** ZnO nanostructures without surface modification are observed as a control used in the experiments.

**Biofunctionalization**


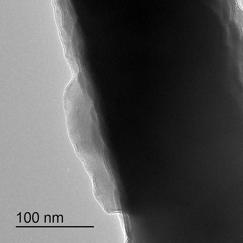

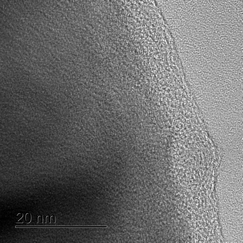


**Fig. S3.** The monolayer resulting after biofunctionalization process is observed on the surface of 1DZnO nanostructures.

**Detection**


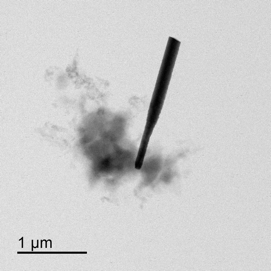
**
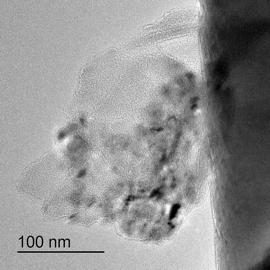
**

**Fig. S4.** The TEM micrographs are observed with an agglomerate of a protein nature consistent with the complex formed by the target and the biofunctionalization process.


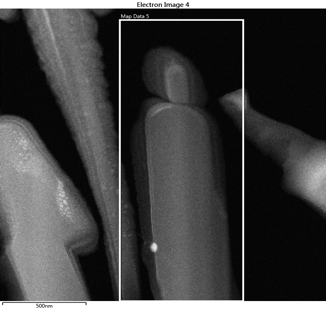

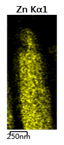

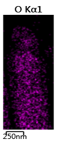

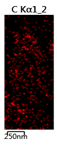

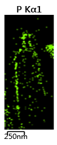

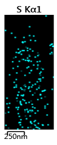

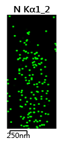

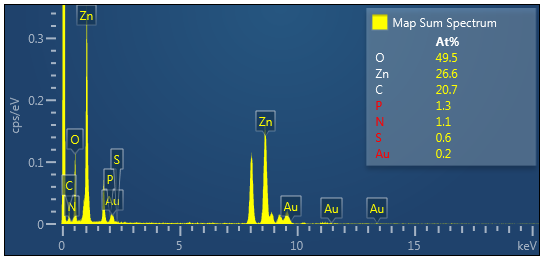


**Fig. S5.** Elemental mapping by EDS of one single nanowire after CYFRA 21-1 detection in PBS.


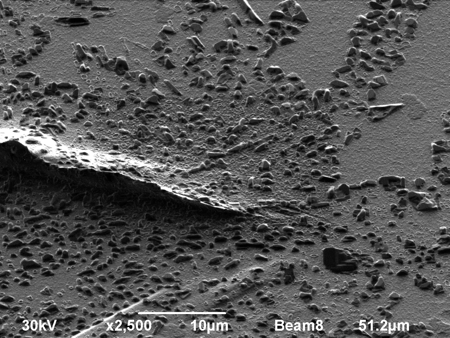


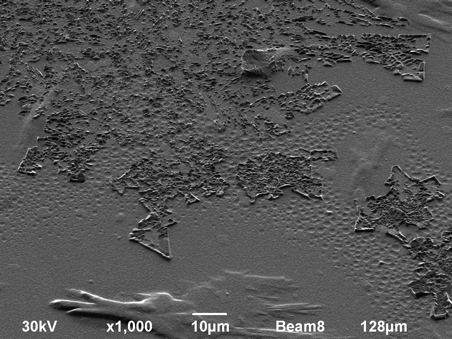


**Fig. S6.** SEM micrographs of artificial saliva adhered to Si substrates.

**

**Fig. S7.** Bovine Serum Albumin (BSA) calibration curve for Bradford protein assays between concentrations from (0 – 30 μg ml^-1^).
