# Supplementary figures and images for: Synergistic Assembly of 1DZnO and Anti-CYFRA 21-1: A Physicochemical Approach to Optical Biosensing
Source: BME Front. 2024 Sep 18;5:0064. doi: 10.34133/bmef.0064 (PMC11408934; doi:10.34133/bmef.0064)

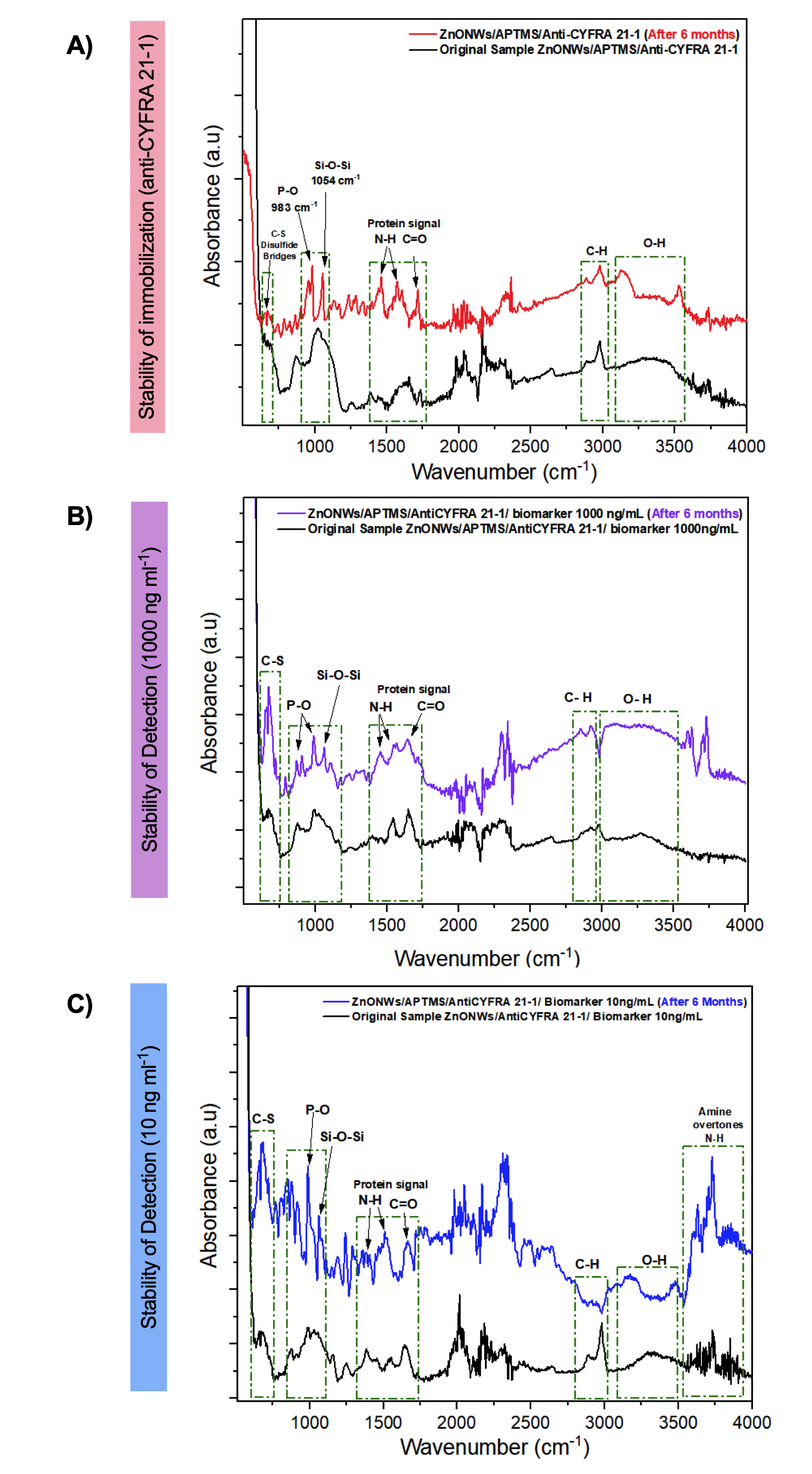

Supplement: Supplementary 1 — Figs. S1 to S7 [file bmef.0064.f1.zip › Fig. S1.tiff]

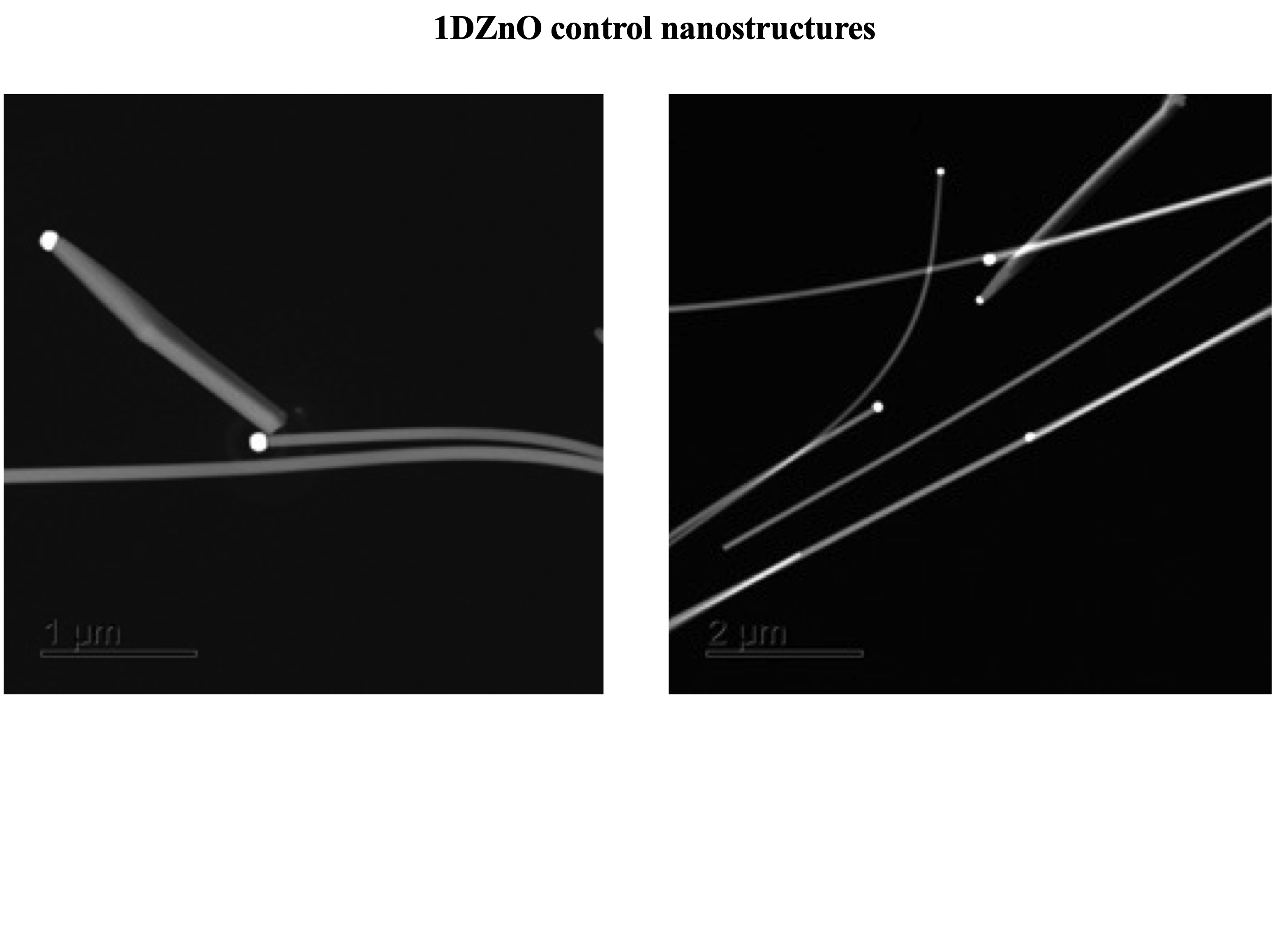

Supplement: Supplementary 1 — Figs. S1 to S7 [file bmef.0064.f1.zip › Fig. S2.tiff]

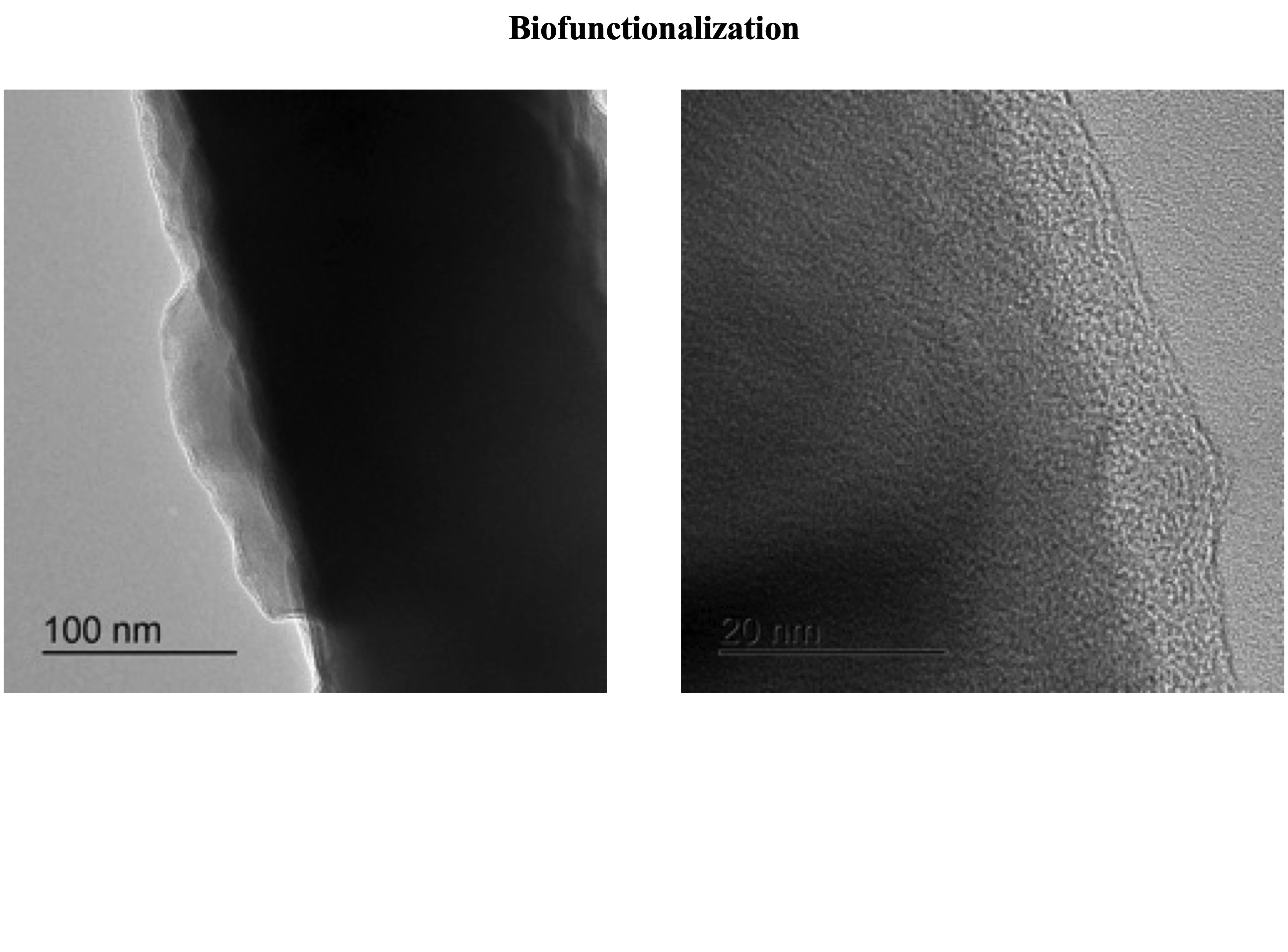

Supplement: Supplementary 1 — Figs. S1 to S7 [file bmef.0064.f1.zip › Fig. S3.tiff]

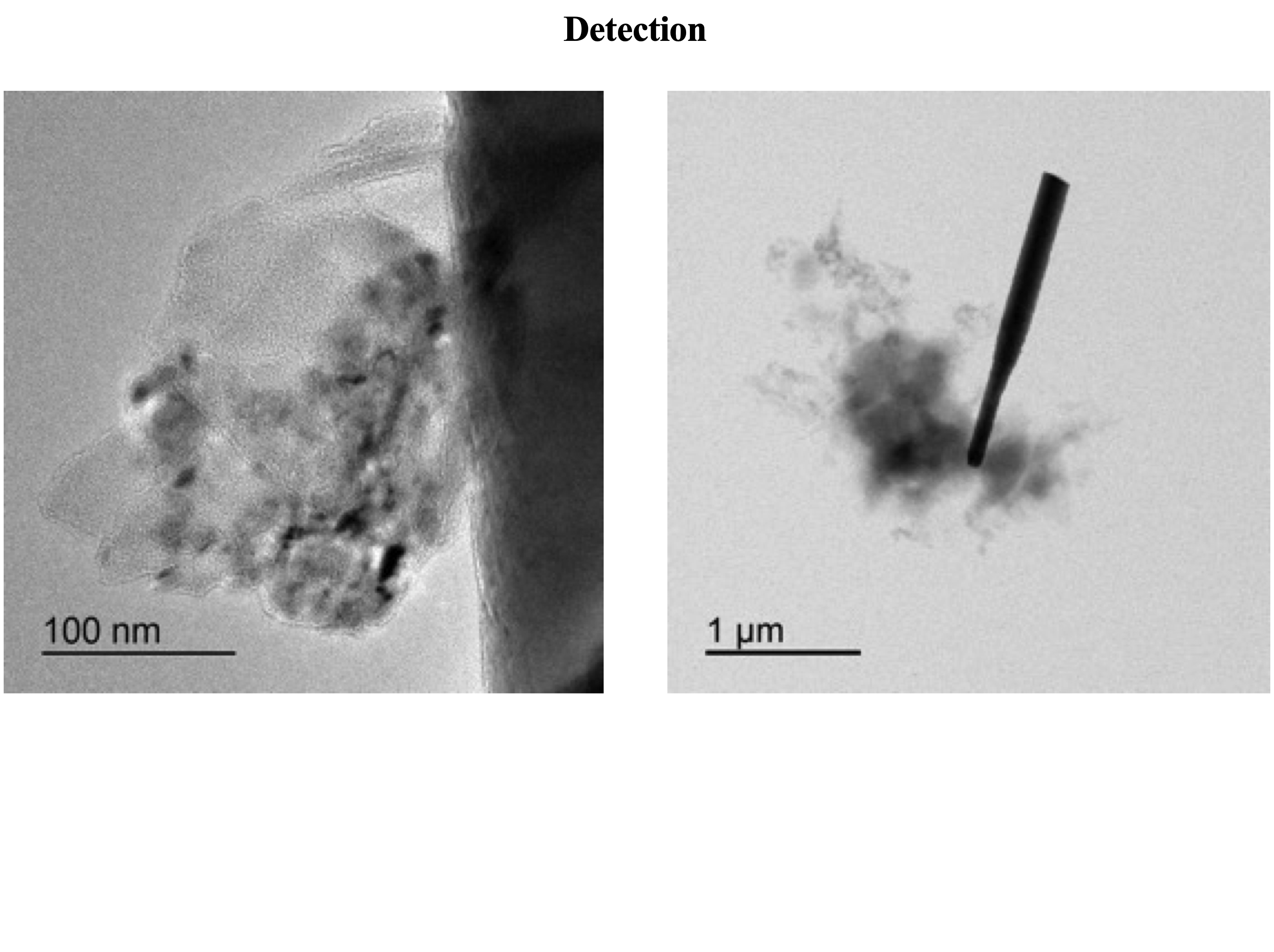

Supplement: Supplementary 1 — Figs. S1 to S7 [file bmef.0064.f1.zip › Fig. S4.tiff]

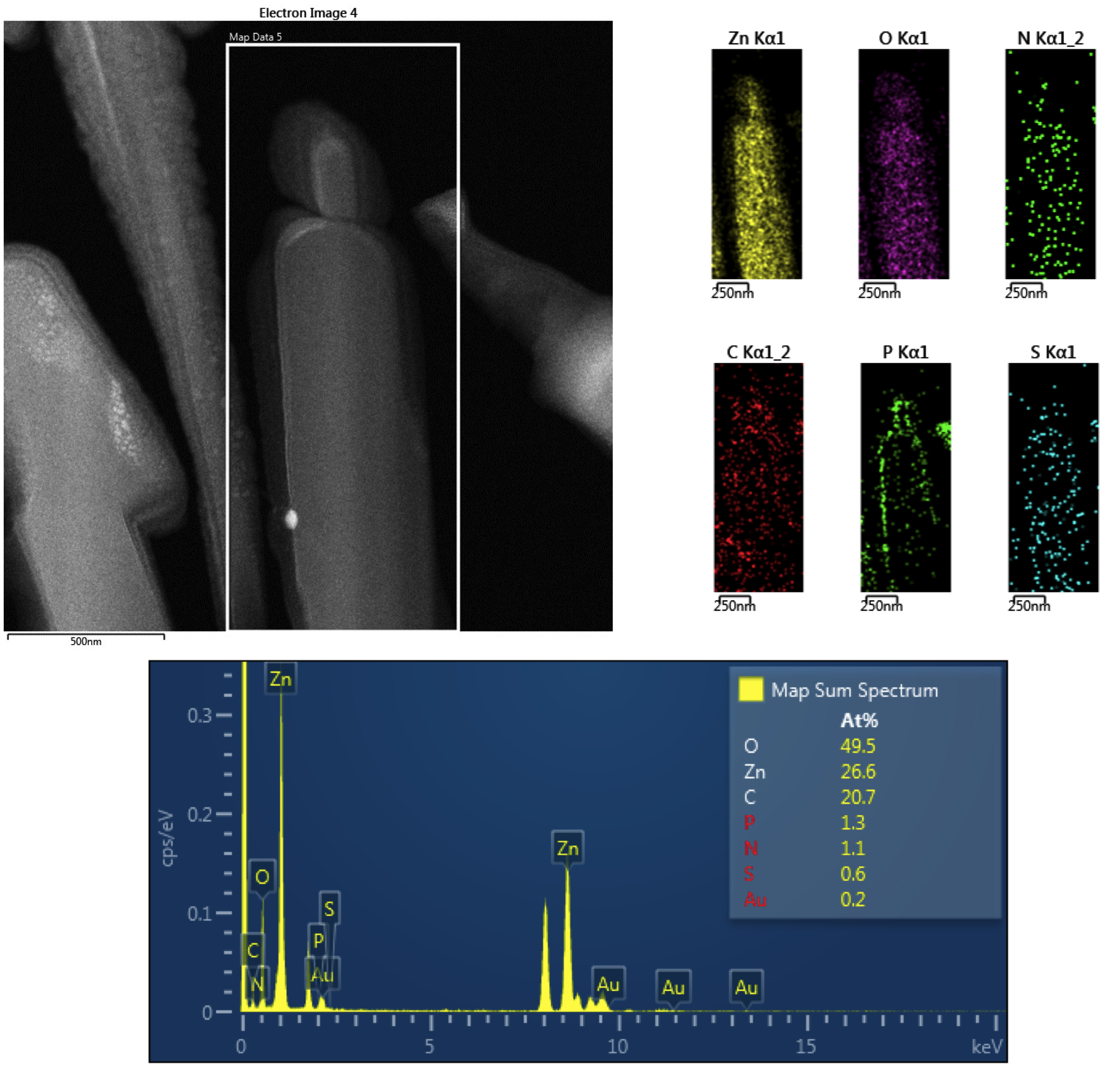

Supplement: Supplementary 1 — Figs. S1 to S7 [file bmef.0064.f1.zip › Fig. S5.tiff]

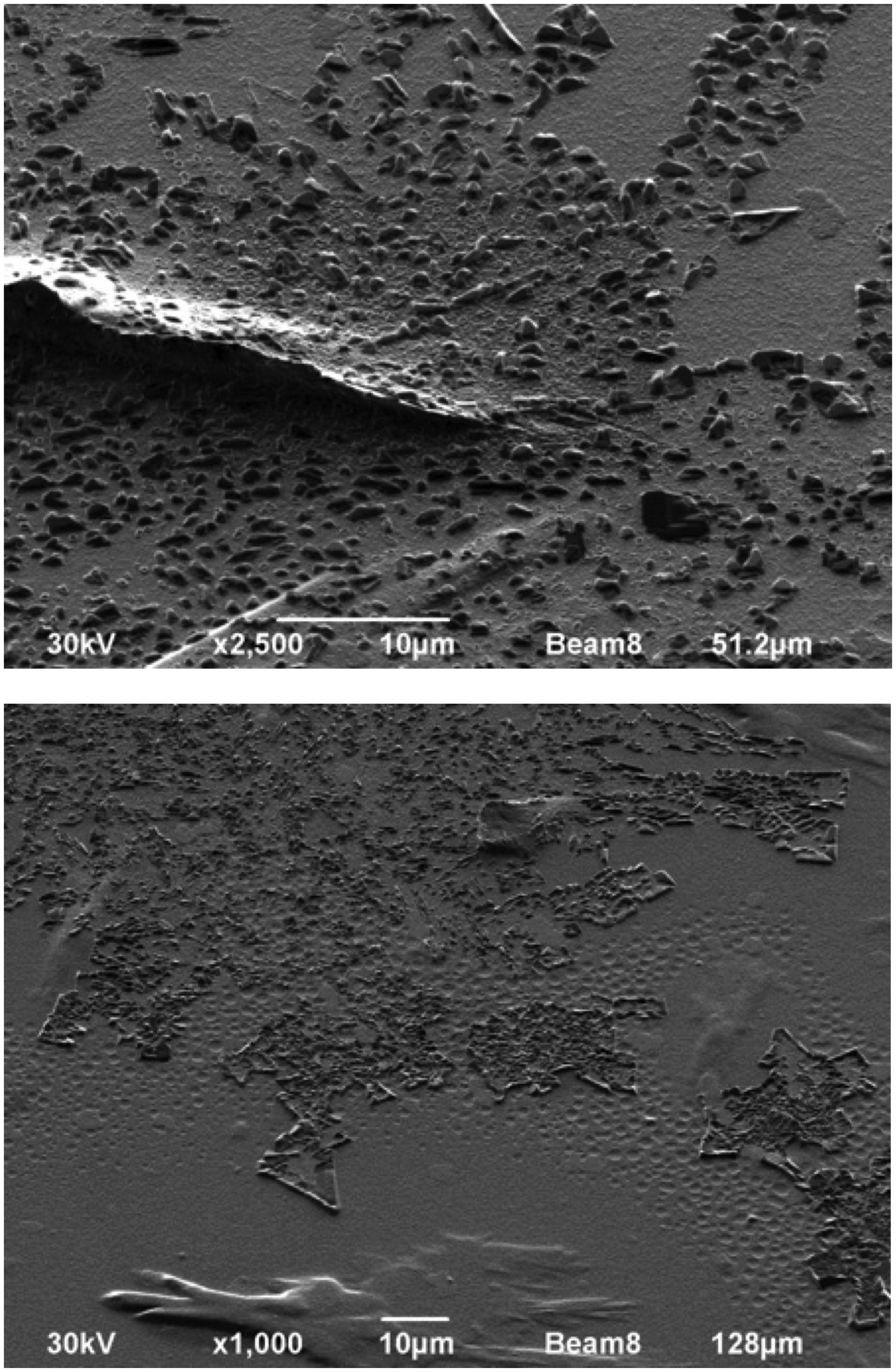

Supplement: Supplementary 1 — Figs. S1 to S7 [file bmef.0064.f1.zip › Fig. S6.tiff]

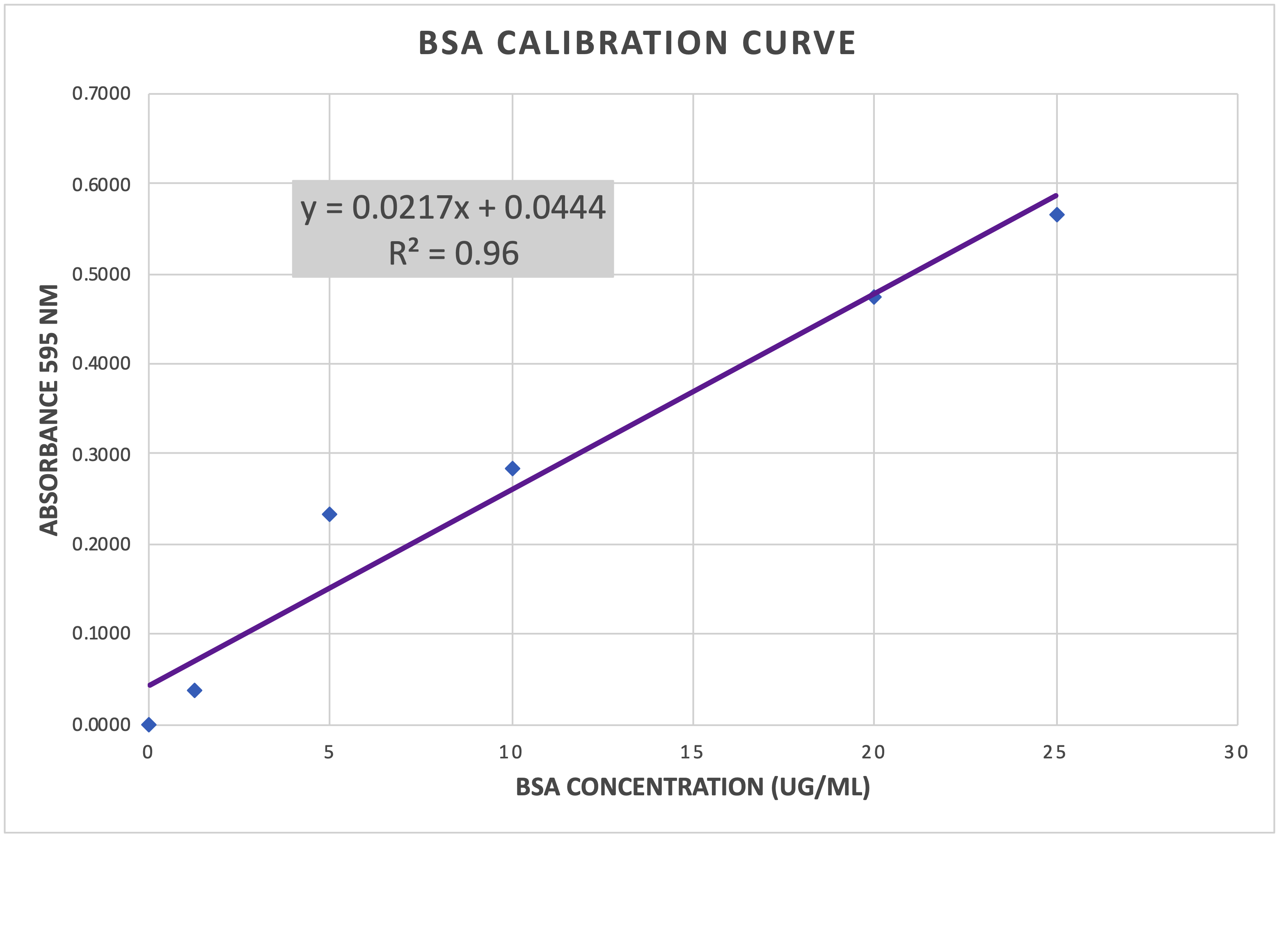

Supplement: Supplementary 1 — Figs. S1 to S7 [file bmef.0064.f1.zip › Fig. S7.tiff]
